# Exploring the Relationships Between Four Aging Ideals: A Bibliometric Study

**DOI:** 10.3389/fpubh.2021.762591

**Published:** 2022-01-21

**Authors:** Ka Lin, Yumei Ning, Ayesha Mumtaz, Hua Li

**Affiliations:** ^1^College of Public Administration, Zhejiang University, Hangzhou, China; ^2^Institute of Policy Studies, Lingnan University, Hong Kong, China; ^3^School of Public Administration, Guizhou University, Guiyang, China; ^4^School of Economics and Management, Huzhou University, Huzhou, China

**Keywords:** aging studies, elderly care, health care, bibliometric, CiteSpace, social policy

## Abstract

When examining research articles on the aging strategies, four ideals (i.e., successful aging, healthy aging, productive aging and active aging) could be explored by conducting bibliometric analyses. For the literature analysis, general information on the four aging ideals was understood through visualization analysis; the intellectual base and research hotspots were intuitively observed. CiteSpace was used as the method to conduct the co-occurrence analysis of keywords in order to obtain research trends and cutting-edge knowledge in the field of aging-related policies. Subsequently, the study revealed the nature of the link between these four aging ideals and disclosed the connection between their fundamental principles. The study ultimately enhanced the understanding of the diverse contexts that have impacted the way in which these ideals influence policy, which has caused dissimilar strategies for policy development. The study also extended the discussion of the definitions of and relationships between these four ideals with the goal of identifying new directions for aging-related practice and providing innovative insights and references for investigators.

## Introduction: Four Aging Ideals

The challenge of having an aging society in the contemporary world creates a need for elderly care and corresponding policy measures. The four ideals of aging (i.e., healthy aging, successful aging, productive aging, and active aging) underline these policy measures (1–3). In the general features, healthy aging as a complex phenomenon involves a lifelong process and depends on the sustained, efficient ability to maintain good health in the elderly. Active aging recognizes the factors affecting individuals' actions in their daily lives. Productive aging stresses the output of the economic and service production of elderly people and successful aging refers to both active aging and healthy aging from the biological, psychological and societal standpoints.

**Figure 1:**
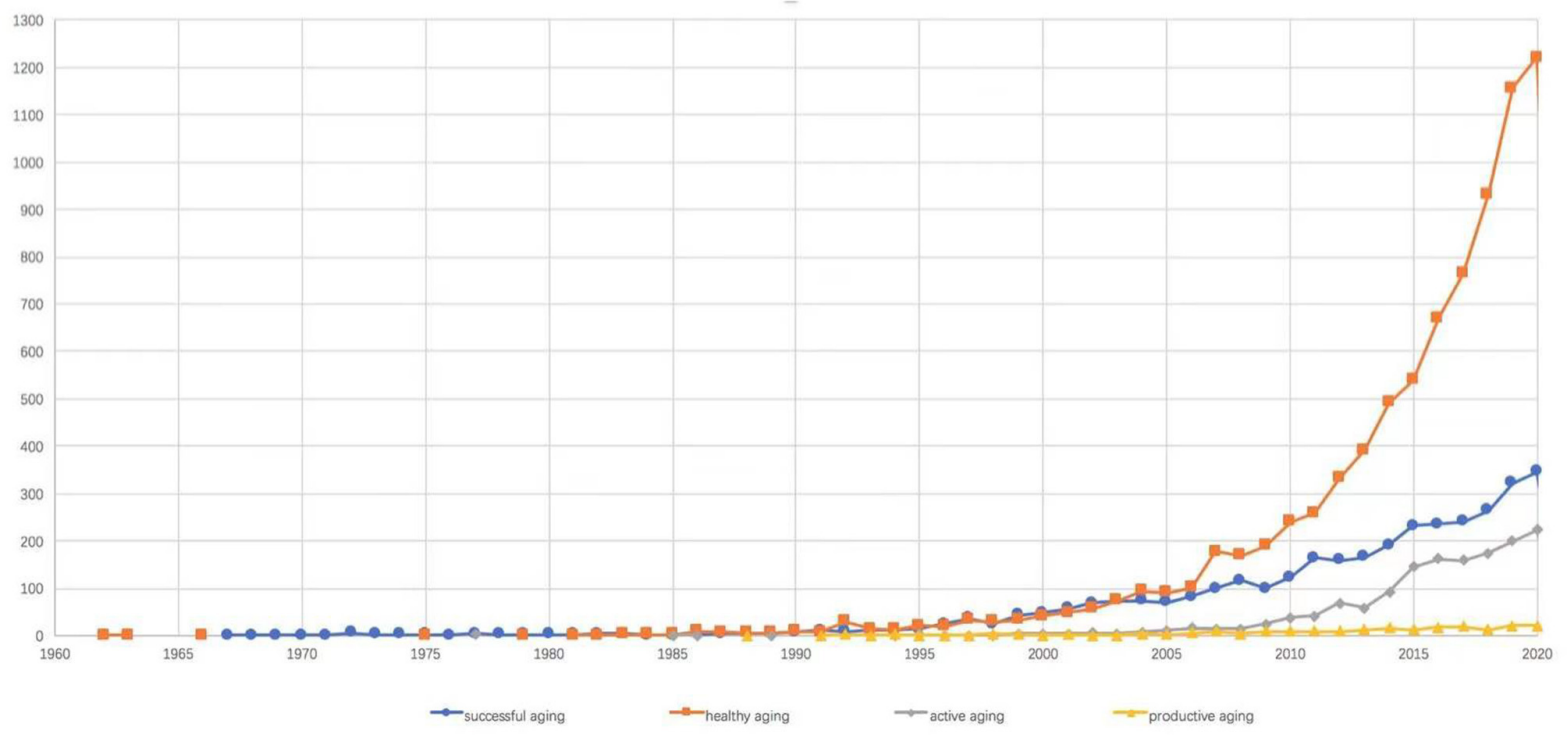
Annual distribution map of aging articles. Data resource: Web of Science, 2021/02/28.

Since each of these ideals has logical grounds for unique policy direction, they all support the theoretical assumptions for different policy paradigms and the guidelines of policy development. The discussion of these ideas began in the 1960s and has grown in scope since the 1990s, as evidenced by an increasing number of articles published in academic journals. According to the evidence collected from the Web of Science, publications covering these ideals were scarce from the 1960s to the 1990s but increased drastically after 2005 (Figure 1). Nevertheless, the popular usage of these ideals in current studies has not revealed their profound logical grounds, especially when the ideals contain diverging viewpoints on aging-related concepts in different countries. From the data obtained for this study, it was evident that there has been no consensus on the definitions or features of these ideals or the relationships between them. Thus, a conceptual and theoretical discussion has become critical to capture their features.

To identify the special features of the ideals, healthy aging can be interpreted as “the process of developing and maintaining the functional ability that enables well-being in older age” [(4); p. 28]. In a narrower sense, healthy aging can be defined as maintaining the elderly in good health and keeping them autonomous and independent for a long period in their later years (5); health conditions in this case refer to both physical and mental health. However, in a broader sense, these can be regarded as the adapting of life and dietary habits to enrich physical and mental health (4, 6). In this context, the ideal of healthy aging supports the guidelines for the promotion of health service policies to improve the quality of life for older people (7).

Active aging means to fully participate in social life and show one's full potential in society. Adopted by the World Health Organization (WHO) in the late 1990s, the term “active aging” was defined as the participation of older persons in activities that contribute to their quality of life and well-being. Walker (8) extended this definition by arguing that active aging refers to the inclusion of older people's activities in the social sphere. Therefore, active aging policies should have two contrasting policy approaches: one with a narrow economic focus on the working sphere of older people, the other focused on their participation in the wider realm of societal activity. Based on this understanding, the concept of active aging could have a function of social inclusion/exclusion.

The ideal of productive aging refers to the participation of older people in different types of work activities and the production of various goods and services necessary for daily living (9, 10). In some circumstances, productive aging is understood to be the capacity and ability of an individual and society to create and reinforce the conditions, opportunities and capabilities of the elderly for productive and meaningful engagement in the economy and society (11). These activities include post-retirement jobs and other productive practices in the industrial, agricultural or other life spheres (12). These activities can be formal or informal, in the voluntary or service sector, for families or the community as a whole.

Successful aging can be measured by examining many aspects of people's personal lives. According to Bowling (13), successful aging refers to the physical, mental and social aspects of elders' lives; Lee et al. (14) take the physical, psychological and social support factors as key elements, with leisure activity as the main indicator of successful aging. Similarly, Hsu (15) measures successful aging by the variables of physical health, independence, living without chronic disease, living with family and receiving emotional care. To test the extent of successful aging, Depp and Jeste (16) adopted physical and psychological health, satisfaction, and social factors as the essential components of successful aging. With the overview of these features, we get the basis for comparison among these ideals.

Nonetheless, in view of the complications and difficulties in these ideals, we feel that the ambiguity in the relation of their definitions is a barrier to comparison. For instance, many scholars often mix active aging and productive aging or use the words “healthy aging” and “successful aging” interchangeably. According to Boudiny (17), the ideals of active aging and healthy aging emphasize the same thing but convey different aspects of the connection between activity and health. Bowling (18–20) also explains the overlapping usage of the active aging and successful aging concepts. A report by Euro Health Net (21) argues that healthy aging is linked to empowerment of the elderly and is thus connected to active and productive aging. Meanwhile, the WHO's ideals of productive and active aging have been regarded as having the potential to “optimize the opportunities for health, participation, and security to enhance the quality of life as people age, and to be incorporated into the WHO's framework by the functions” [(7); p. 12].

Although some authors believe that the term “healthy aging” is preferred over “successful aging,” many others regard these two terms as overlapping. The mixed view of these issues is due in part to the nature of studies on aging since the research relates to comprehensive disciplines. Psycho-social scientists may define it as the potential for one's behavioral and psychological features, whereas medical practitioners define healthy aging by incorporating the functioning of physical and mental health. For social and policy analysis, some scholars complain that these ideals are often interchanged, making it difficult for researchers to render a clear idea of their connections (1). Minkler and Holstein (22) maintain that the ideals of successful, healthy and active aging contain implicit normative standards. This creates the need to relate the social norms of elderly care to different policy orientations while distinguishing one from the other and makes it easy to categorize them by technical measures to conduct a comparative analysis.

## Research Framework and Methods

When there appears vague boundaries between the ideals and complicated classifications of their meaning creates a challenge for researchers, research comparing the ideals to be necessary in both theoretical and practical terms. However, the scarcity of research on the conceptual framework obstructs the potential to execute theoretical studies and evaluate their policy implications. The relationships between the four ideals should be explored by developing conceptual comparative studies, undertaking theoretical reconstruction with certain methodological underpinnings. To undertake this research task, this study used a comparative analysis of the links between the four ideals and applied a bibliometric analysis of the selected literature for comparison. The bibliometric features of the research objects were revealed by the application of Citespace software as a measurement tool (23).

As one of the most significant tools for the scientific mapping of literature, Citespace is widely applied in health psychology (24) and economic research (25). The software uses co-citation analytical method and the pathfinder algorithm and has been widely used in the research of bibliometric analysis all over the world. It has the advantage of measuring the literature for the collection of articles in a specific subject field. Keyword analysis helps to examine the hotspots and research topics at a certain time, whereas burst words, found among all keywords, serve as important indicators when forecasting emerging trends and frontier topics (26). The visualization maps that CiteSpace creates generally involve nodes and links; the nodes represent the elements of analysis, such as country, keyword, cited reference, author and institution, and note the frequency of occurrence or citation.

By using the keyword search method through bibliometric analysis on the CiteSpace software, the study collected its research data by searching for journal articles on the well-known Web of Science database. The standard for article selection was set by the keywords “successful aging,” “healthy aging,” “active aging,” and “productive aging.” The study collected the data of selected articles by using the “combined keywords” search method. This method established the research data basis for the comparative analysis and began the exploratory journey by defining the four aging ideals, then broadening the areas of comparison. The data was defined by keyword search through the titles, abstracts and subject keywords of the articles in the period of 1990 to 2020. The type of document was set as “research article” and the language was set as “English.” The articles were then downloaded and converted to TXT format and the abstracts were extracted from the journal publications.

## Results

With the foundational data for this study established, the data was imported into the Citespace V software to calculate the selected terms for the analysis and combine the searches, which resulted in 4,715 articles. Among those articles, there were 2,509 articles containing the keywords “successful aging AND healthy aging,” 771 articles on “successful aging AND active aging,” and 209 on “successful aging AND productive aging.” By combining three keywords: “successful aging AND healthy aging AND active aging,” we found 119 articles for the study. However, a combined search of four keywords: “successful aging AND healthy aging AND active aging AND productive aging,” did not bring up any results (see Table 1). In the following sections, we will discuss the relationships between successful aging and the other three aging ideals. The term “successful aging” was used as the center of the comparison, partly because the paragon of successful aging captures the broad elements of all four ideals, involving the interaction between physical health, functional capabilities, life engagement and so on. This allowed a wider scope of issues to be covered in this study and enriched the scope of analysis on aging related policies.

**Table 1:** The combined search of four aging ideals.

**Datasets**	**Topic**	**Topic search**	**Citation expand**	**Combined**
1	Successful aging + healthy aging	166	2,343	2,509
2	Successful aging + active aging	31	740	771
3	Successful aging + productive aging	10	199	209
4	Healthy aging + active aging	84	596	680
5	Healthy aging + productive aging	6	151	157
6	Healthy aging + active aging + productive aging	1	108	109
7	Active aging + productive aging	4	157	161
8	Successful aging + healthy aging + active aging	6	113	119
9	Successful aging + healthy aging + productive aging	0	–	–
10	Successful aging + active aging + productive aging	0	–	–
11	Successful aging + healthy aging + active aging + productive aging	0	–	–

### Successful Aging and Healthy Aging

The relationships between these two ideals are complicated. For instance, Bowling and Iliffe (27) argue that the discussion on successful aging can refer to the biomedical, broader biomedical, social and psychological factors, thus making it possible to create a multi-dimensional model. This complication requires scholars from the fields of social science, psychology and demographic and gerontological science, as well as social policy and care experts, to contribute to aging studies. This creates a demand for an all-encompassing and coordinated strategy, analyzed by EU-co-supported ventures (2004–2007), that considers age with reference to all areas of society and engagement. Thus, with “successful aging” as the central keyword, we examined its relations with the corresponding aging ideals.

When analyzing the data in the CiteSpace software, we used the combined search feature with the keywords “successful aging” and “healthy aging” and found 2,509 documents (see Table 1). Among these documents, the most frequently used keywords concerned ideals that can be categorized into three main clusters: health, mental health and the social aspects of life. The first set of keywords referred to “health,” which is one of the conditions necessary for a successful life for the elderly (28). Thus, in the study of healthy aging, the factors of physical activity, physical function, nutrition and longevity are all involved (29, 30). In the keyword analysis, some physical illnesses, such as Alzheimer's disease, obesity, cardiovascular disease and epidemiology, were frequently reflected in the resulting articles.

**Figure 2:**
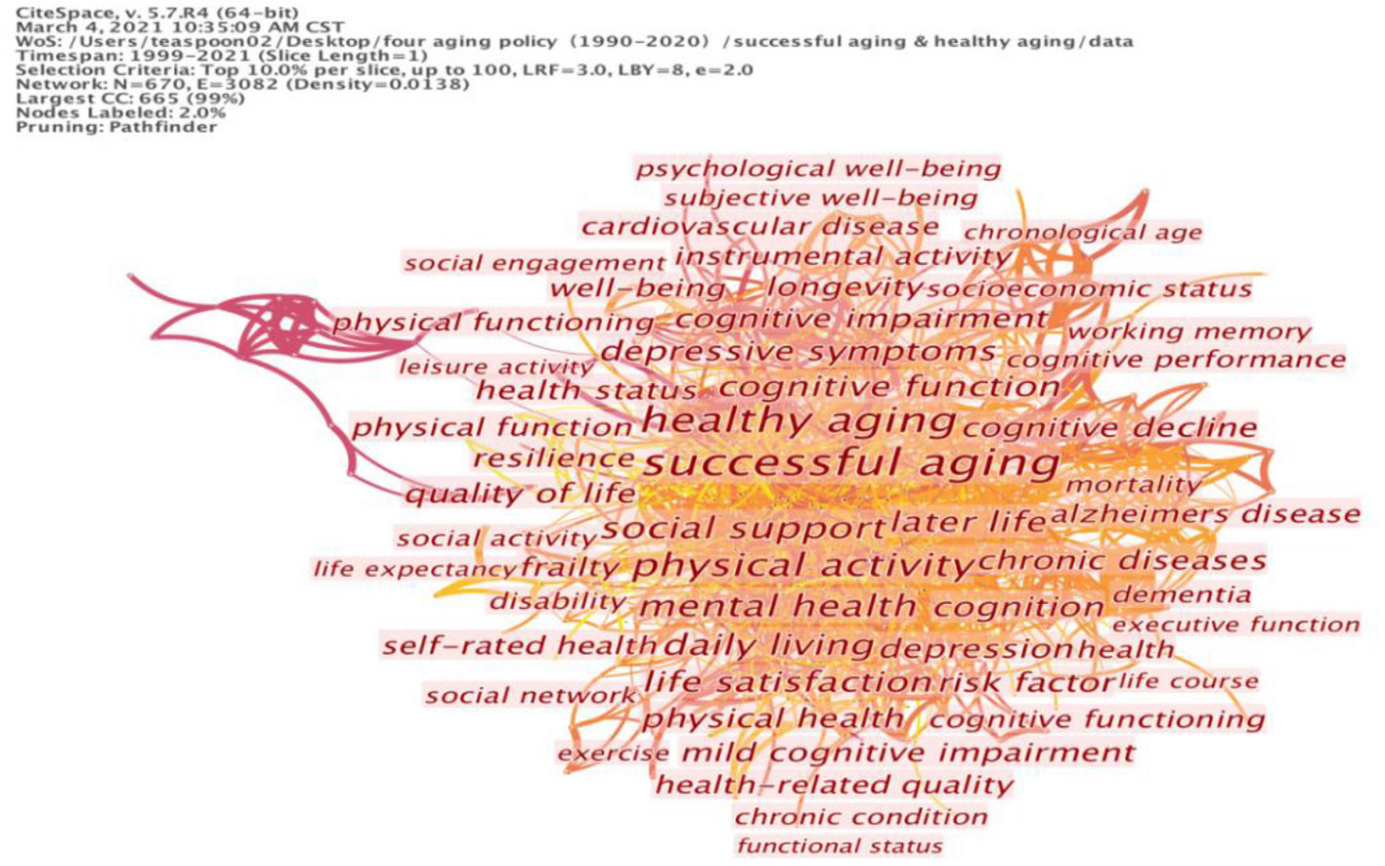
Cluster map of keywords on successful aging and healthy aging.

The second cluster covers the subject of mental health, with the keywords “cognitive function,” “depressive symptoms,” and “cognitive functioning.” By maintaining these basic keywords, we can also extend the keywords “mental” and “self-rated health” to the elderly's expectation of health and the individual's subjective well-being. The indicators of subjective well-being can include happiness, satisfaction, attachment and a sense of belonging, which are central concepts in successful aging. For this reason, we should also highlight the significance of social factors that support successful and healthy aging. Cluster three involves the social aspects of life: “social support,” “daily living,” “social activities,” “social network” and “social engagement.” These keywords are shown in Table 2; the corresponding cluster map is shown in Figure 2. This directs the tendency of successful aging to comprise the subjective factors and provide a broader view of a healthy life by including success in social networking, which can be used to assess the extent of subjective well-being.

**Table 2:** Successful aging and healthy aging.

**Keyword**	**Freq**	**Centrality**	**Keyword**	**Freq**	**Centrality**
**Cluster 1: health**					
Successful aging	507	0.04	Alzheimer's disease	49	0.04
Healthy aging	307	0.04	Health-related quality	48	0.04
Physical activity	197	0.03	Well-being	47	0.01
Social support	142	0.06	Cardiovascular disease	44	0.1
Health status	56	0.04	Frailty	61	0.01
Physical function	54	0.1	Self-rated health	54	0.04
Exercise	26	0.01	Physical functioning	43	0.02
Health	43	0.04	Physical health	69	0.04
**Cluster 2: mental health**					
Mental health	128	0.04	Cognitive functioning	44	0.08
Daily living	120	0.03	Working memory	29	0.02
Later life	104	0.03	Chronic condition	30	0.02
Cognitive function	104	0.05	Active aging	40	0.02
Life satisfaction	99	0.04	Mortality	39	0.01
Depressive symptoms	88	0.04	Dementia	35	0.01
Cognition	77	0.06	Mild-cognitive impairment	62	0.01
Cognitive impairment	75	0.03	Cognitive performance	34	0.03
Depression	73	0.04	Disability	34	0.02
Quality of life	71	0.04	Subjective well-being	31	0.02
Chronic diseases	67	0.09	Psychological well-being	30	0.04
Cognitive decline	67	0.01			
Risk factor	65	0.05			
**Cluster 3: social aspects of life**					
Social activity	31	0.02	Life course	23	0.02
Resilience	64	0.02	Social network	29	0.01
Social engagement	27	0.03	Functional status	20	0.04
Longevity	59	0.05			
Leisure activity	23	0.04			
Life expectancy	23	0			
Executive function	22	0.01			
Instrumental activity	50	0.02			

From the above-mentioned evidence, we can infer some problems in the links between successful aging and healthy aging. The dominant role of health is evident in the literature; the articles mostly concentrate on the issue of health (31), whereas the central concepts of successful aging are seen less frequently. From a health perspective, the keywords regarding psychological and mental health are presented as health indicators rather than measurements of individuals' subjective well-being. Thus, if we recall what Bowling and Iliffe (27) proposed, successful aging could refer to the biomedical model (predominantly comprising the variables of physical and mental health functioning) and the socio-psychological model (that emphasizes life satisfaction, social functioning and psychological resource indicators); the keyword analysis of the literature seems to incline more toward the biomedical model than the socio-psychological model.

The analysis also showed that the impact of social factors on both successful and healthy aging is rarely addressed. When viewing the keyword map, societal factors and social support are barely reflected in the results, with the exception of the keywords “family relations.” Many influencing social factors of successful aging are missing from the keyword list, particularly the issues of social policy and services for the elderly. The lack of societal factors in successful aging research indicates that previous studies mainly focused on clinical orientation instead of public services. The collected papers seem to ignore the social dimension of successful aging; thus, social support should be emphasized in its discussion.

### Successful Aging and Active Aging

**Figure 3:**
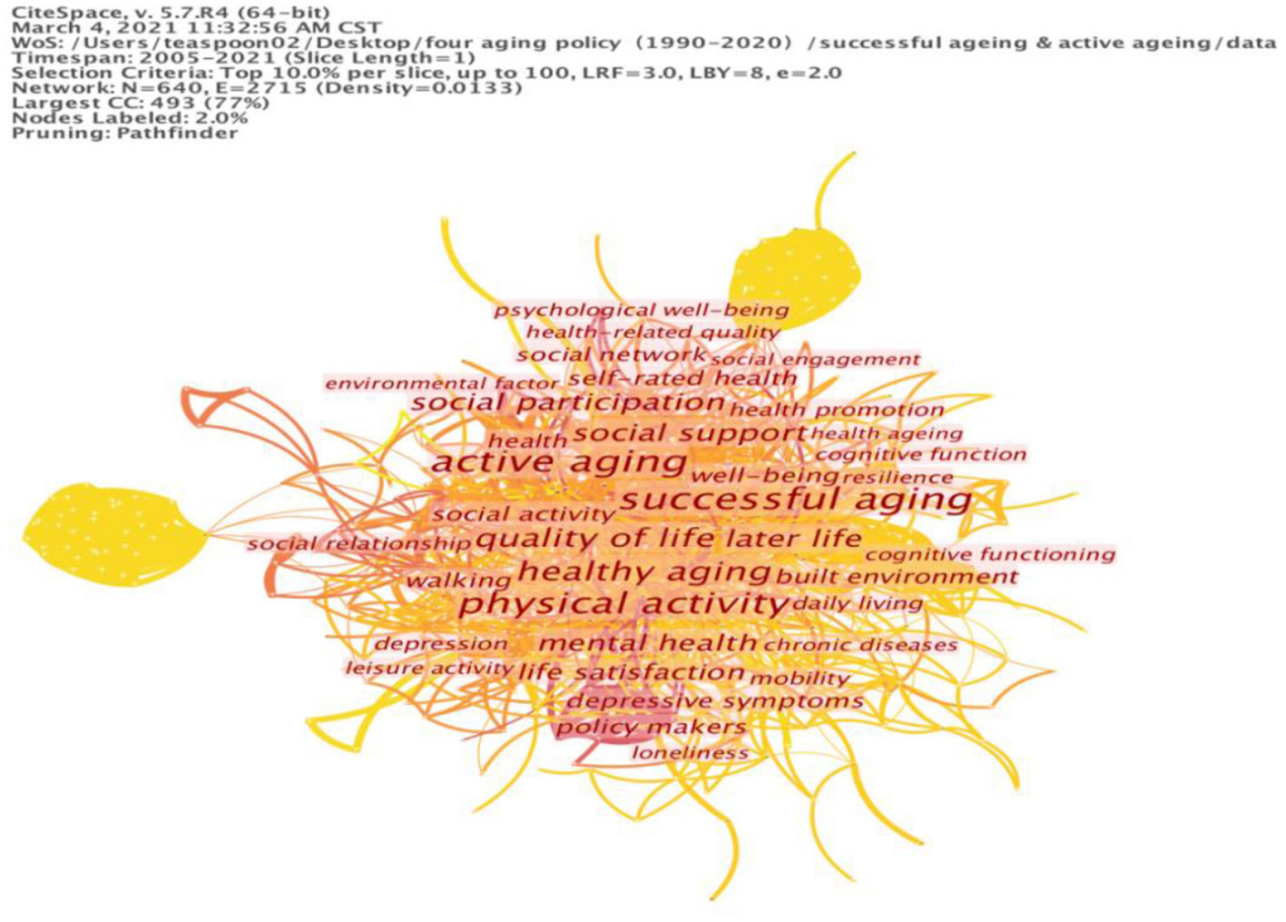
Cluster map of keywords on successful aging and active aging.

To explore the relationship between successful aging and active aging, the combined keyword search resulted in a total of 771 articles, as shown in Table 3; the cluster map of the keywords is shown in Figure 3. Active aging is comprised of individual physical activity and the avoidance of disease and disability, providing the foundation for a high standard of physical and cognitive functioning. However, the WHO (32) states that physical functioning should not be the only issue under the umbrella of healthy aging; the focus should also be on developing personal capacity and ability. Berlin et al. (33) underline sport and exercise-based activities, acknowledging them as the predictors of successful aging among older American women. Thus, to promote active aging, it is important to allow older people to find their own ways to navigate life's changes (34). Additionally, the social activities of participation and empowerment also require perspectives on active aging that support successful aging.

**Table 3:** Successful aging and active aging.

**Keyword**	**Freq**	**Centrality**	**Keyword**	**Freq**	**Centrality**
**Cluster 1: daily life actions/quality of life**					
Quality of life	54	0.06	Leisure activity	11	0.03
Daily living	14	0.04	Life satisfaction	26	0.07
Mobility	13	0.01			
**Cluster 2: social participation**					
Social support	39	0.02	Social participation	37	0.03
Social network	15	0.01	Social engagement	10	0.08
Social relationship	13	0.03			
**Cluster 3: health related actions**					
Physical activity	91	0.04	Health	16	0.03
Health promotion	14	0.01	Cognitive function	12	0.07
Psychological well-being	11	0.02	Cognitive functioning	11	0.01
Depression	13	0.03	Chronic diseases	12	0.07
Loneliness	11	0.02	Healthy aging	59	0.06
Mental health	29	0.09	Health-related quality	12	0.01
Self-rated health	17	0.03	Depressive symptoms	17	0.06
Walking	17	0.02	Health aging	10	0.01
Well-being	19	0.02	Resilience	12	0.03
**Cluster 4: other keywords**					
Active aging	122	0.07	Successful aging	125	0.06
Later life	34	0.04	Environmental factor	10	0.01
Built environment	17	0.01	Policy makers	17	0.03
Aging population	17	0.02			

With these components, the data calculation reveals three clusters of keywords. The first cluster is based on keywords focusing on daily life actions, including “quality of life,” “life satisfaction,” “daily living,” “mobility” and “leisure activity.” The second cluster is comprised of the keywords related to social participation, including “social support,” “social activity,” “social network,” “social participation,” “social relationship” and “social engagement.” The third cluster is the largest and contains keywords centring on health actions, such as “physical activity,” “health,” “health promotion,” “cognitive function,” “psychological well-being” and “cognitive functioning.” The third cluster also contains keywords relevant to special diseases and illnesses, for instance: “depression,” “chronic disease” and “loneliness.” The cluster map of the keywords is shown in Figure 3.

With respect to daily life, we can see the use of the reflected keywords for active aging in regard to social activities, which convey a more comprehensive message than just “healthy aging.” By using the successful aging ideal as the center, we can observe its usefulness in understanding daily life actions, from a view of human interaction in the daily activities and approach it from practice angles. For instance, leisure activities are an effective indicator of the lifestyles of the elderly, which should be well-elaborated. This implies the need for specific studies that promote an improved quality of life. In this context, the WHO defines active aging in terms of the participation of older persons in activities that enhance their quality of life and well-being [(7), p. 12].

Beyond daily activities, two indicators are useful in the research on successful aging according to the data analysis: participation and empowerment. Regarding social participation, active aging is not just about the individual's good physical health but also their participation in social activities and making a positive contribution to society (35). The issues of social support should be essential to social networking and empowerment, but the literature only mentions the active engagement of individuals. Accordingly, the elderly should be encouraged to integrate into mainstream society and develop their own social projects and cultural activities.

Still, health is the precondition of active aging. The analysis of current research shows that the understanding of active aging is emphasized in the meaning of physical health and activities. For instance, Stenner et al. (36) argue that the policy of active aging should encourage families and citizens to make “personal efforts to adopt positive personal health practices at all stages of life.” This background induces Walker (8, 37), who deviates from the health aspect of aging embodied in policy documents, emphasizes the inclusion of social activity. This extends the scope of the focus on active aging to include successful aging.

### Successful Aging, Productive Aging, and Active Aging

To get a more comprehensive understanding of these relationships, we shall examine the links between the ideal of productive aging and the ideals of active and healthy aging. In Herd (38) view, for instance, successful aging includes an element that relates to productive aging; according to Ferreira (34), the best incentive to encourage older people to continue their work is to enhance active aging. Once they are engaged in productive work and contributing to society, they may have a sense of successful aging (39). Thus, Butler and Gleason (40) proposes the participation of the elderly in paid work or voluntary work in the community's care-related facilities. This feature distinguishes productive aging from healthy aging (see Table 4). This results in the scarcity of research studies on the productive aging because of the weak connection with health care, thus creating an urgent need to clarify the relationship between these ideals.

**Table 4:** Successful aging and productive aging.

**Keyword**	**Freq**	**Centrality**	**Keyword**	**Freq**	**Centrality**
**Cluster 1: daily life activities**					
Care	7	0.01	Later life	22	0.37
Life course	12	0.29	Intergenerational relationship	4	0.3
Life satisfaction	4	0.05	Active role	3	0.02
Proactive behaviors	3	0.02			
Active aging	8	0.02			
**Cluster 2: health activities**					
Health	3	0.02	Healthy aging	5	0.04
Successful aging	58	0.3	Mental health	5	0.18
**Cluster 3: production activities**					
Older workers	7	0.11	Work	3	0
Working adults	4	0.03	Productive aging	12	0.18
Aging workforce	3	0	Occupational future time perspective	3	0
Work ability	6	0.13	Job performance	3	0.01
**Cluster 4: social activities**					
Ageism	6	0.08	Civic engagement	4	0
Social relation	3	0	Social support	5	0.01
Social engagement	3	0.07	Social relationship	3	0
Social activity	3	0.01	Positive relationship	3	0
Personhood	4	0.21	Subjective age	5	0

**Figure 4:**
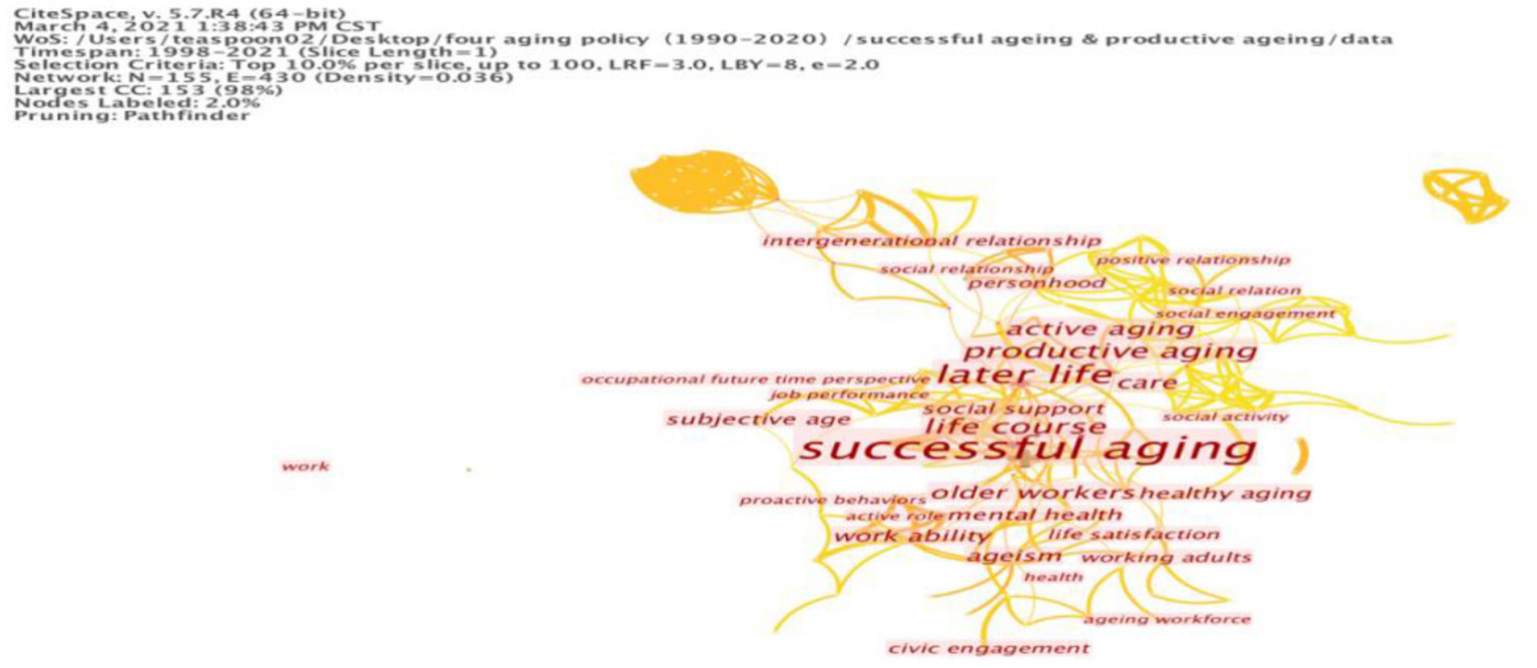
Cluster map of keywords on successful aging and productive aging.

Based on this view, we used the same method to explore the connection between active aging and productive aging in relation to successful aging. By keyword search, we also obtained 209 articles combining “successful aging” and “productive aging.” The cluster map of these combined terms is shown in Figure 4, categorized the keywords into four clusters: daily life activities, health activities, productive activities, and social activities. The first cluster refers to daily life activities. Daily life activities and the identified terms illustrate a group of words concerning quality of life. Cluster two is associated with health factors, but health-related terms are scarce in the “productive aging” group. Thus, the term “productive aging” has broad relevance to various influencing factors of aging but is not limited to healthy aging, as productive aging mainly refers to production (i.e., formal and informal, paid and unpaid) and is partly associated with successful aging.

Cluster three refers to included productive activities including factors of “worker”, “workforce” and “work ability”. There is a popular assumption that productive aging is similar to and has a close relationship with active aging (41); however, productive activities hardly seem related to successful aging due to the traditional image of the elderly as retirees who enjoy their leisure time and reduced workload. Nevertheless, the gains from work are the source of satisfaction, which engenders a sense of success. Thus, when making policy, we should emphasize the productivity of the elderly as a valuable asset rather than a burden to society.

The fourth cluster of the keywords analysis refer to social activities, social relations, social engagement, and social support. In a broader view, the social factors of productive aging can be measured by the various indicators suggested by Lum (42), including market activities (employment), non-market activities (grandchild care and family care) and both formal and informal social activities (helping community members) that contribute to one's quality of life and life satisfaction. Thus, the elderly's activities should be viewed as contributions to productive forms of aging. In this vein, successful aging includes productive aging to improve the individual's well-being and quality of life, together with a sense of happiness and life satisfaction. To some extent, productive aging promotes the market value of formal and informal work; this may give the elderly a feeling of achievement.

**Figure 5:**
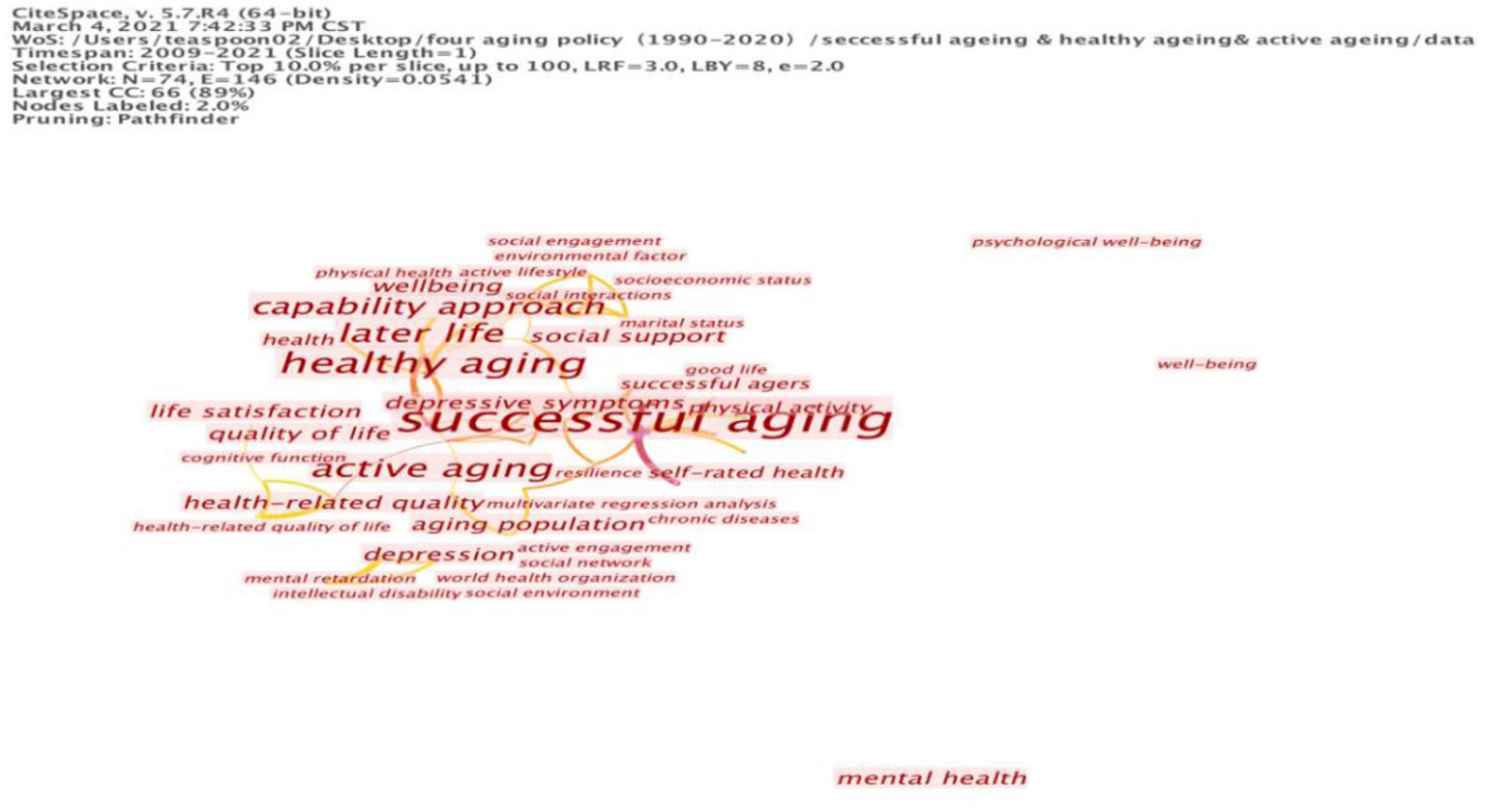
Cluster map of keywords on successful aging AND healthy aging AND active aging.

In the search of the combined terms “successful aging AND healthy aging AND active aging,” we found 119 articles (see Table 1). Combining the search results in Table 5 resulted in three main clusters of keywords as shown in Figure 5. Among the identified clusters, we categorized the keywords into “health performance,” “social performance” and “quality of life.” We were not surprised to see the indicators of health performance as the largest cluster. We also confirmed the value of social factors to successful aging, as the term “success” for the elderly does not only include health or activities, but contribution to society. This can produce a strong feeling of social belonging. We also paid attention to the third cluster: quality of life. Indeed, when examining the features of successful aging, we should underscore the meaning of life satisfaction and quality of life with inclusive activities in social networking and happiness.

**Table 5:** Successful aging AND healthy aging AND active aging.

**Keyword**	**Freq**	**Centrality**	**Keyword**	**Freq**	**Centrality**
**Cluster 1: health performance**					
Healthy aging	15	0.17	Psychological well-being	2	0
Chronic diseases	2	0	Mental health	4	0
Physical health	2	0.02	Health-related quality	4	0.14
Depression	4	0.2	Intellectual disability	2	0.01
Health-related quality of life	2	0.01	Mental retardation	2	0.01
Depressive symptoms	4	0.05	Resilience	2	0.02
Cognitive function	2	0.05	Successful aging	42	0.54
Health	3	0	Self-rated health	3	0
Physical activity	3	0			
**Cluster 2: social performance and participation**					
Active aging	11	0.34	Social environment	2	0
Social engagement	2	0.03	Social support	4	0.06
Active engagement	2	0.03	Social interactions	2	0.03
Active lifestyle	2	0.06	Social network	2	0.03
**Cluster 3: quality of life**					
Quality of life	4	0.18	Life satisfaction	4	0.06
Later life	11	0.02	Socioeconomic status	2	0.06
Marital status	2	0.02	Good life	2	0
Well-being	2	0	Well-being	4	0
Capability approach	8	0.16	Successful agers	3	0
Elderly people	4	0.17	Environmental factor	2	0
World-Health Organization	2	0.02			

We remained curious about any common elements, so we put these ideals together with different orientations. These different orientations seem far apart, and their complicated relationships have rarely been explored. Thus, we used the combined search with the keywords “successful,” “healthy,” “active” and “productive aging.” Unfortunately, this search did not return any articles. Meanwhile, in the common view, the production force of the elderly is typically low-income, therefore it has a weak connection with successful aging. Nonetheless, these factors are not presented in contrast but are distinguished from one another and explore their relations through keyword comparison. Reflected in the academic literature, these common views influence the number of publications on this issue. Thus, we developed a better understanding of the features of these concepts as they relate to different aspects of aging strategies, illustrating the divergent areas of emphasis in policy creation.

## Conclusion and Implications

This paper systematically studies four aging concepts and compares the subjects, their features and policy implications by using the keyword analysis method. Among the ideals, healthy aging and successful aging support the studies on the bio-clinical model of aging for an individual's physical well-being and psychological state. The active aging ideal and productive aging standards support a social view of aging, requiring individuals to be able to hold a productive job (21). To understand their connections, the successful aging paradigm is positioned in the center of the analysis of these different ideals, highlighting the differences between them. The rich context of these ideals implies that active aging stresses the importance of health and exercise, but social cooperation in various organizations is not emphasized. Thus, any discussion of aging should go beyond health and nursing to social support and productive activities to benefit the elderly in the context of other aging ideals. This study highlights the complicated features and, more importantly, the missing elements in aging-related strategies.

Through the four-model comparison, we discovered that healthy aging is the dominant term to recognize the positive health outcomes in later life. This dominance prohibits other factors (such as successful aging) from broadening their influence through bibliometric analysis. Meanwhile, the economic function of the productive aging ideal is not emphasized; thus, aging-related policies should be made in a broader context. Quality of life and elders' sense of happiness and satisfaction should be highlighted; the ideals of active aging and productive aging also contribute to life satisfaction and the best possible health (43, 44). This study uncovered the meaning of these various ideals beyond healthy outcomes to aspects of well-being.

At the operational level, this study sets successful aging as the standpoint for comparison, revealing the distance from one ideal to another. From this standpoint, we can capture the concept of productive aging, making participation in formal and informal work the source of feelings of success. Thus, happiness indicators and quality of life are discussed in connection with health policies and economic positions. This connects successful aging to social equality and empowerment. The active aging and productive aging ideals also support certain orientations of policy proposals, as these orientations lead to different aging ideals with rich variables in development. It should be noted that many of these ideals have previously been ignored, thus this study of bibliometric analysis highlights some overlooked issues in aging-related policies.

From these observations, we can assess the policy implications of this research. The comparative analysis of this study informs us of the need to strengthen the study of social factors that improve the social base of aging studies. Researchers should also broaden their classical “mental and physical health” view of aging ideals by incorporating more factors to construct a comprehensive concept (45). This establishes a better foundation for policy analysis and the development of more realistic and effective measurements of aging strategies. For instance, the WHO (4) prescribes that in order to accomplish healthy aging, experts should work from an intersectional point of view, connecting the elderly, family and society, and creating activities “that enable the elderly to smoothly make transition into the next stage of their lives,” However, the current literature reveals that these ideas are mainly set at the individual level of activities rather than the societal level. Thus, to construct a comprehensive concept of aging ideals, raising the level of studies on quality of life for the elderly would be helpful when debating elderly care policies.

In all, this analysis may provide a theoretical basis and a framework for policy studies aimed at building an elderly-friendly society. It may also help us better understand the problems related to aging, find coping measures and foster a sound and inclusive social environment for the elderly. It encourages the inclusion of elders in activities and services through society's support systems with an obligation to enhance social welfare. However, we have a need to align studies based on healthy aging and active aging with successful aging and productive aging. It is advised to adopt inclusive policies through the active aging approach, which can still provide a sound basis for different countries throughout the world to respond to the challenges resulting from an aging population.

## Data Availability Statement

The original contributions presented in the study are included in the article/supplementary material, further inquiries can be directed to the corresponding authors.

## Author Contributions

KL contributed to the main conceptual ideas, design, research framework, and paper drafting. YN take the responsibility of data collection, analysis, and calculation. AM involved in the drafting process and including full manuscript revision. HL aided in interpreting the references, results, contributed in revision, and proof reading. All authors contributed to the final revision of the manuscript.

## Funding

This research was supported by the research funds from National Social Science Fund of China (No. 19ASH016), Chinese post doc Foundation, and other resources.

## Conflict of Interest

The authors declare that the research was conducted in the absence of any commercial or financial relationships that could be construed as a potential conflict of interest.

## Publisher's Note

All claims expressed in this article are solely those of the authors and do not necessarily represent those of their affiliated organizations, or those of the publisher, the editors and the reviewers. Any product that may be evaluated in this article, or claim that may be made by its manufacturer, is not guaranteed or endorsed by the publisher.
